# 

**Published:** 1781-03

**Authors:** 


### PROMOTIONS.

Sir Richard Jebb, Bart. M. D. F. R. S. to be phyſician in ordinary.—Thomas Keate, Eſq; to be

*. Later experiments, however, render it very doubtful whether so large a dose was ever repeated if ever given; the stomach always containing such a quantity of air as to inflame the phosphorous, and the fraction of a single grain having induced most alarming symptoms when swallowed in a bolus.

be ſurgeon in ordinary; and R. Halifax Eſq; to be apothecary in ordinary to the Prince of Wales. —Dr. Richard Proctor, late apothecary to the hoſpital in North America, to be phyſician to the army under Sir H. Clinton.—John Whitehead, M. D. to be phyſician to the London Diſpenſary, in the room of John Kooſtray, M. D. deceaſed.—M. De Laſſonne to be firſt phyſician to the King of France, in the room of the late M. Lieutaud.—M. Tiſſot, phyſician at Lauſanne, to be profeſſor of phyſic at Pavia, in Auſtrian Lombardy. The Emperor allows him to ſpend ſix months of the year in Switzerland.

### DEATHS.

*Feb.* 18.—At Milton near Sittingbourn in Kent, Mr. Iſaac Grayling, ſurgeon and apothecary.

28.—At Annan in the county of Dumfries in Scotland, Mr. John Hall, ſurgeon and apothecary.

*March* 8.—At Derby, Mr. Thomas Brown, ſurgeon and apothecary.

23.—In Lawrence Pountney-lane, London, Mr. Lawrence, apothecary

24.—In Curſitor-ſtreet, Chancery-lane, Mr. James Skene, ſurgeon.

### SECTION IV.

### MONTHLY CATALOGUE.

1. AN affectionate Tribute to the Memory of the late Dr. John Fothergill. By *W. Hird*, M. D. 4to. London.

2. A critical Enquiry into the ancient and modern Manner of treating the Diseases of the Urethra, with an improved Method of Cure. By *Jesse Foot*, one of the company of surgeons in London, and privileged practitioner from the college at St. Petersburgh. The third edition, 8vo. London.

3. Specifique contre la Goutte, eprouvé et publié, par *M. Emerigon*, procureur du roi, en la jurisdiction royale, et au siege general de l'Amiranté du Bourg St. Pierre. *i. e.* a Specific for the Gout, tried and made public by *M. Emerigon*, &c. 8vo. Paris, 48 pages.

This remedy, which it seems has long been employed by the natives of the Caribbee islands in gouty complaints, is nothing more than a strong infusion of gum guaiacum in rum; the dose is a table spoonful every morning fasting. The author, who is not a medical man, extols the the efficacy of this medicine in his own caſe. He deſcribes the regimen and mode of life requiſite for the due operation of the medicine, and theſe are ſuch as are alone ſufficient to moderate, and even to prevent the moſt dreadful effects of the gout. His rules on this ſubject conſiſt in adviſing the patient to uſe exerciſe, to avoid an indolent and luxurious manner of living, to drink milk two hours after the medicine, to eat ſparingly at dinner, to avoid ſuppers, to drink Burgundy wine diluted with water, to go to bed at night, and riſe at five in the morning; and laſtly, to avoid cold.

The author aſſerts, that before he began the uſe of this medicine, he was ſubject to frequent acid eructations and other ſymptoms of indigeſtion, from all which he is now free.

The names of ſeveral perſons are mentioned who have experienced the good effects of this remedy, which is likewiſe ſpoken of as a uſeful medicine in cholic, ulcers, ſciatica, catarrhal and rheumatic complaints.

4. Anatomicarum Annotationum Liber primus. De Nervorum Gangliis & Plexubus, auctore *Antonio Scarpa* in Mutinenſi Archigymnaſio Anatomes & Chirurgiæ Profeſſore, Noſocomii Militaris, & Legionum ſereniſſimi Mutinenſium

Ducis Chirurgo Primario. 4to. Mutinæ 1779, 149 pages.

This volume is divided into four chapters; in the firſt the author deſcribes the ſtructure of ganglions; in the ſecond he endeavours to aſcertain their uſes, and theſe he ſuppoſes to conſiſt chiefly in collecting, mixing, and dividing the nervous fibres; in the third he treats of the uſe of the ſpinal ganglions, brachial plexuſes, and other nervous plexuſes of the human body; and in the laſt ſpeaks very fully of the conſent of parts. The work, which ſeems to be extremely accurate with regard to anatomical deſcription, is illuſtrated by two excellent copper-plates, each of which contain ſeveral figures.

5. Precis d'une Nouvelle Theorie ſur les Maladies Chroniques, particulierement les purulentes, ſcorbutiques, nerveuſes, dartreuſes, et generalement ſur toutes celles qui proviennent de la decompoſition du ſang. Par *M. Baſtays*, M.D. Medecin de l'hopital militaire de la Ville de l'Orient. *i. e.* An Account of a New Theory of Chronic Diſeaſes, particularly purulent, ſcorbutic, nervous, cutaneous, and in general all ſuch complaints as are occaſioned by a decompoſition of the blood. By *M. Baſtays*, M. D. &c. 12mo. Amſterdam 1780, 345 pages.

This

This performance is not founded on facts, but ſeems to be merely the fruit of the author's imagination.

6. Memoire ſur les moyens à employer pour s'oppoſer aux ravages de la Variole; par *M. Maret*, M. D. ſecretaire perpetuel de l'academie de Dijon. *i. e.* A diſcourſe on the means to be employed for preventing the ravages of the Small pox. By *M. Maret*, M. D. &c. 8vo. Paris, 160 pages.

7. Experiences nouvelles ſur les proprietés de l'alkali volatil fluor, par *M. Martinet*, Curé de Soulaines. *i. e.* New experiments on the properties of the volatile alkali fluor. By *M. Martinet*, Curate of Soulaines. 8vo. Paris, 1781.

8. Calendarium Medicum ad uſum ſaluberrimæ facultatis Medicæ Pariſienſis, &c. Ann. 1781. 12mo. & 4to. Paris.

9. Voyage Mineralogique fait en Hongrie et en Tranſylvanie, par *M. de Born*; traduit de l'Allemand, avec quelques notes, par M. Monnet, inſpecteur general des mines de France, &c. *i. e.* Mineralogical travels through Hungary and Tranſylvania, by *M. de Born*, tranſlated from the German, with notes by M. Monnet, inſpector general of the mines in France, &c. 12mo. Paris, 1780.

10. Re-

10. Reflexionen und Erfahrungen für Bürger, Geistlicke und junge Aerzte. *i. e.* Reflexions and observations for citizens, clergymen, and young physicians. 8vo. Dusseldorp, 1780.

In this periodical paper, the author, who seems to be a physician of considerable knowledge and genius, delineates in the most striking colours the different prejudices, errors, and abuses that prevail in Germany with regard to physic. His observations on these subjects are equally applicable to many other countries. His remarks are altogether popular, without being diffuse, prejudiced, or theoretical, and are therefore very agreeable and instructive to read.

11. *Ernst. Anton. Nicolai,* D. A. D. &c. Wissenschaft der Krankheiten. *i. e.* Pathology, by *E. A. Nicolai,* M. D. professor of physic in the University of Jena in Saxony. 6th and last volume. 8vo. Halle, 758 pages.

The author has comprized his system of pathology in 6 volumes 8vo. His chief merit seems to be, that of being a good compiler. His work contains some few prejudices and erroneous opinions; but, these excepted, it may be considered as the best work of the kind extant, though the author has not brought us a step ftep farther in pathological fcience than we had attained twenty years ago.

12. Abhandlungen aus der Naturgefchichte practifchen Arzneykunft und Chirurgie aus den fchriften der Haarlemer und anderen Holländ-ifchen Jefell Schaften. *i. e.* Effays and differta-tions on natural hiftory, practice of phyfic and furgery, extracted from the tranfactions of the Harlem and other learned focieties in Holland, with copper-plates. 8vo. Leipfic.

This work is intended for phyficians and fur-geons; all papers on mathematics, natural phi-lofophy, &c. being omitted, and fuch only re-tained as are interefting to the medical reader. The different articles are judicioufly tranflated and abridged. We could wifh to fee a fimilar publication in Englifh.

13. Traité fur les Gonorrhées. Par *M. Guerin*, ancien chirurgien de marine, maitre en chirur-gie à Rouen. *i. e.* A Treatife on Gonorrhoeas, by *M. Guerin*, furgeon at Rouen, formerly a navy furgeon, 12mo. Paris 1780, 90 pages.

14. Meteorographie, ou Art d'obferver d'une Maniere commode et utile les Phenomenes de l'Atmofphere, &c. avec des planches en taille-douce. *i. e.* Meteorography, or the Art of ob-

ſerving in a convenient and uſeful Manner the Phænomena of the Atmoſphere, &c. with two copper-plates. By *M. Changeux.* 8vo. Paris, 1781, 42 pages.

15. Obſervations ſur l'Operation Céſarienne à la ligne blanche, et ſur l'uſage du forceps, la tete arretée au detroit ſuperieur; par *M.F.A. de Leurye*, profeſſeur et demonſtrateur des accouchemens aux ecoles royales de chirurgie à Paris. *i. e.* Obſervations on the Cæſarian Operation at the Linea Alba, and on the Uſe of the Forceps when the head is obſtructed at the brim of the Pelvis; by *M. F. A. de Leurye*, profeſſor of midwifery in the royal ſchools of ſurgery at Paris. 8vo. Paris, 105 pages.

